# Dermatologic management of a deep graphite foreign body in the dorsal hand: ‘Hand surgery for the dermatologist’

**DOI:** 10.1016/j.jdcr.2026.03.038

**Published:** 2026-03-27

**Authors:** Brittani Remé, Emily Deehan, Ata S. Moshiri, Daniel L. Fischer

**Affiliations:** a Department of Dermatology, Baylor College of Medicine, Houston, Texas; b Dr. Kiran C. Patel College of Osteopathic Medicine, Nova Southeastern University, Fort Lauderdale, Florida; c Dermatopathology Section, Ronald O. Perelman Department of Dermatology, NYU Langone Health, New York, New York; d SINY Dermatology, New York, New York; e Department of Dermatology, Ronald O. Perelman Department of Dermatology, NYU Langone Health, New York, New York

**Keywords:** cutaneous foreign body, dermatologic surgery, dorsal hand anatomy, granulomatous inflammation, graphite foreign body, pencil-core granuloma, procedural dermatology, ultrasound imaging

## Introduction

Penetrating injuries from pencils and graphite-containing materials are frequent during childhood. Retained fragments can lead to the formation of localized subcutaneous “graphite balls” or “pencil granulomas,” which may remain inert or develop a granulomatous response years to decades later after initial injury.^1^ While many such lesions are referred to orthopedic or hand surgery, dermatologists trained in surgical techniques can safely remove selected cases when proper anatomic planning and technique are employed. This report demonstrates dermatologic management of a deep graphite foreign body of the dorsal hand.

## Case presentation

**Fig 1:**
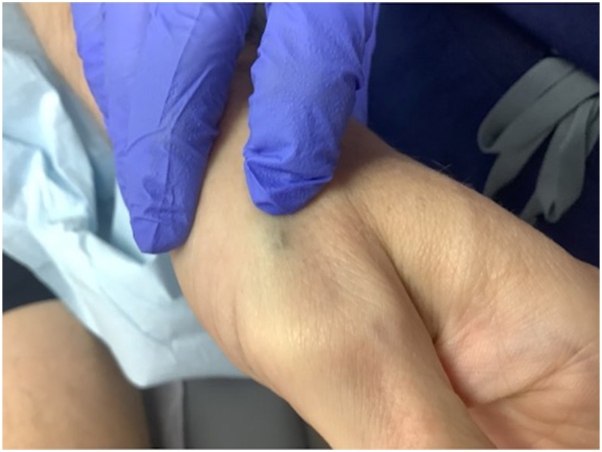
Clinical presentation of a dark blue subcutaneous nodule along the extensor pollicis longus tendon.

A 40-year-old man presented with a soft, mobile subcutaneous nodule measuring approximately 1.5 cm overlying the extensor tendon of the left thumb (Fig 1). The lesion had been present since age 8 after an accidental self-inflicted injury with a lead pencil. The patient noted no pain, functional limitation, or history of infection but sought removal for cosmetic reasons.

**Fig 2:**
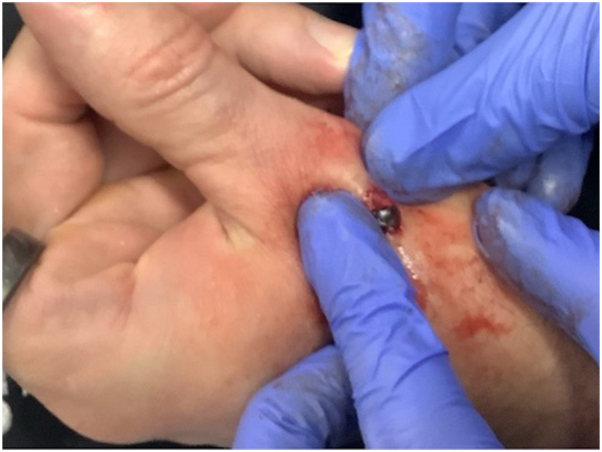
Graphite foreign body adjacent to the extensor pollicis longus tendon prior to complete excision.

On examination, the lesion was a well-circumscribed, dark-blue nodule that translated with active thumb extension, consistent with attachment to the extensor pollicis longus tendon (Fig 2). Under local anesthesia, a 1.6-cm fusiform ellipse incision was made with a No. 15C blade over the dorsal thumb. Sharp dissection was carried through the dermis to the level of the paratenon, where the lesion was identified as adherent to the extensor tendon sheath. Fine-toothed Adson forceps were used to gently grasp the exposed graphite sphere, and blunt dissection was performed to delineate its interface with the paratenon and superficial tendon fibers. With controlled traction to define the plane of attachment, delicate iris scissors were used to sharply release fibrous adhesions, allowing separation from the underlying tendon while preserving tendon integrity. A discrete graphite sphere measuring approximately 5 mm in diameter was removed in its entirety. The wound was closed in layers using 5-0 polyglactin 910 for the deep dermis and 5-0 nylon for the epidermis, followed by petrolatum ointment and a light compressive dressing.

**Fig 3:**
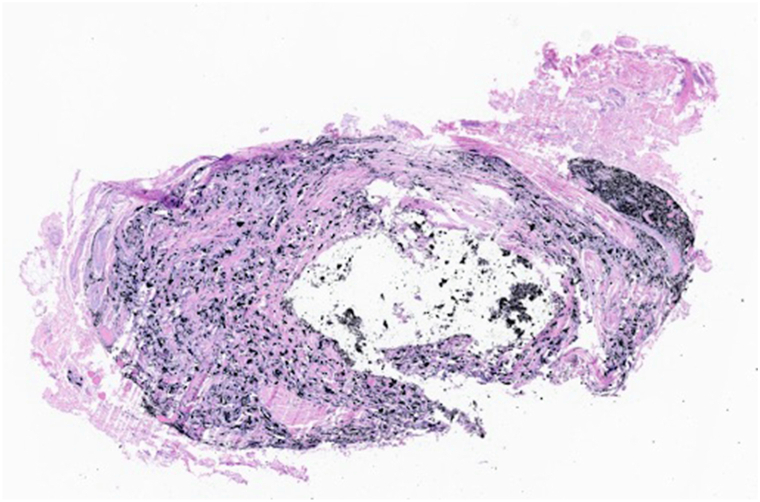
Scanning magnification (H&E, 20×) demonstrates a well-circumscribed granulomatous infiltrate surrounding fine and coarse fragments of black, foreign material.

**Fig 4:**
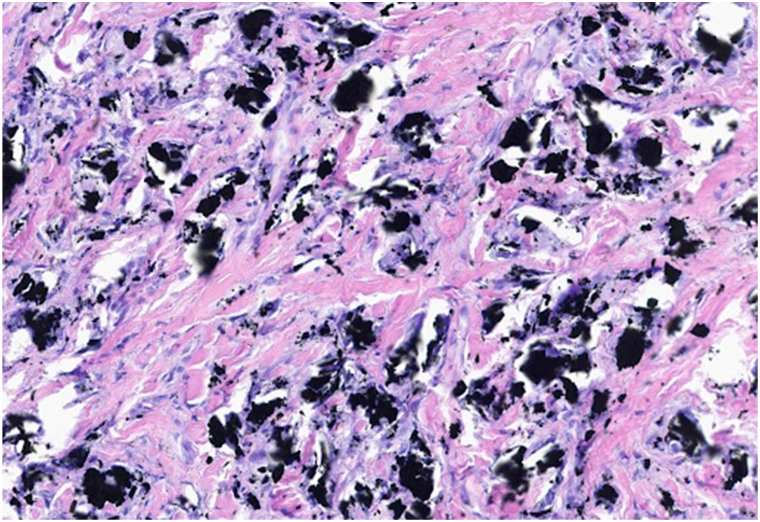
Higher power magnification (H&E, 200×) highlights histiocytes with granuloma formation surrounding the exogenous material.

Histopathologic examination revealed a granulomatous inflammatory infiltrate composed of histiocytes and multinucleated giant cells surrounding fine and coarse black refractile material consistent with graphite (Fig 2, Fig 3, Fig 4). At 2-week follow-up, the wound had healed without complication, with preservation of full range of motion and no evidence of tendon injury.

## Discussion

Retained graphite fragments from childhood pencil injuries can remain asymptomatic for decades but may later present as enlarged pigmented nodules or foreign-body granulomas.^1^ Approximately 38 cases have been reported in the literature, providing a modest but important foundation for understanding the clinical and histologic features of graphite-related granulomas. The lesions are commonly mistaken for cysts, benign tumors, or malignant melanoma, making awareness important for dermatologists when evaluating hand lesions. Although many deep soft-tissue lesions are referred to orthopedic or hand surgeons, dermatologic surgeons can manage selected cases when anatomic factors are considered. Dermatologic training includes extensive experience with excisional surgery, and fellowship-trained Mohs surgeons routinely perform procedures involving the hand and peritendinous tissues, supporting their ability to safely manage lesions adjacent to the paratenon and extensor tendons.

The dorsal hand contains a soft-tissue envelope overlying the extensor tendons, paratenon (connective tissue around the tendon), dorsal veins, and superficial sensory nerves lying beneath the dermis.^2^ Lesions that move with tendon excursion, as seen in our case, suggest adherence to the extensor mechanism and require careful dissection to avoid injury. Protection of the paratenon is crucial, as disruption may lead to impaired tendon gliding, decreased range of motion, and increase the risk of perioperative infection or tenosynovitis. Incisions are best oriented parallel to Langer’s lines, and dissection should be performed in a layered manner to expose the foreign body while maintaining the integrity of the tendon surface.^2^ Our case used a blunt dissection technique to avoid trauma to surrounding structures.

Preoperative imaging may assist with surgical planning. Graphite has been variably reported as radiolucent or radiopaque, depending on its composition and small fragments may be hard to visualize on plain radiographs. Most foreign bodies demonstrate hyperechogenicity with posterior acoustic shadowing or reverberation from surface reflectivity, making ultrasound an optimal imaging choice. In older injuries, ultrasound may reveal a hypoechoic halo surrounding the foreign body, reflecting fibrinous exudate, granulation tissue, or capsule formation, which can aid in identification.^3^ When lesion depth and mobility are well characterized on examination, surgical excision can be performed without preoperative imaging.

Surgical intervention should not be delayed in symptomatic or complicated cases. If a foreign body is clearly visible or doesn’t need removal, imaging isn’t needed, otherwise, X-ray, computed tomography, or ultrasound are appropriate options.^4^ In the absence of imaging, a careful preoperative assessment along with minimal incision is appropriate. Careful dissection is necessary to avoid neurovascular injury. Postoperative management should include wound irrigation, confirmation of foreign body removal in its entirety, and appropriate closure technique, including sutures or steri-strips as needed.^4^ Antibiotic prophylaxis is not routinely administered; however, the patient should be monitored for adverse reactions, including infection, pain, or functional impairment.^4^ We recommend follow-up within 2 weeks postsurgery to ensure appropriate healing and recovery.

Histopathology of retained graphite fragments is representative of a chronic inflammatory response mounted by the body or granulomatous reaction. This presentation consists of macrophages, multinucleated foreign body giant cells, and varying amounts of fibrotic tissue.^5^ The graphite ball material itself appears as gray or black refractile particles within the granuloma.^5^ On gross examination, past cases have described lesions as blue or pigmented nodules.^1^ Overall, our case reinforces the known pattern of foreign-body granuloma formation from retained graphite and underscores the need to consider this etiology when evaluating pigmented cutaneous nodules.

## Conclusion

This case highlights the role of dermatologic surgeons in effectively managing deep foreign bodies of the hand, combining precision, anatomical awareness, and cosmetic closure techniques. Understanding of the dorsal hand’s soft tissue layers, particularly the extensor tendon and paratenon, guides safe incision placement and controlled dissection. Collaboration with hand surgeons may be warranted for complex or functionally critical lesions, but selected cases, such as long-standing graphite nodules, are well within the dermatologist’s surgical domain.

## Conflicts of interest

None disclosed.
